# The Nurses' Resolution

**Published:** 1920-07-10

**Authors:** 


### THE MATRONS' AND SISTERS' DEPARTMENT.

### THE NURSES' RESOLUTION.

The resolution acknowledging Sir Henry Burdett's "great services to the nursing profession," which Sir Everard Hambro, Miss Skillman, R.R.C., and Miss Clara Fisher moved, seconded and supported at the annual meeting of the Royal National Pension Fund for Nurses on June 23, would have given keen pleasure to Sir Henry had he lived to hear it passed. For it quietly points to a fact that is not appreciated by every nurse. Sir Henry's service to nurses was a belief in their power to save for their old age when no one else believed in it; an addition to their savings of £20,000 followed by procuring a handful of rich men to guarantee an initial fund of that amount, which was afterwards made an absolute gift. A provision was thus created through the Fund for the old age of nurses at a time when the profession was miserably paid, and had no friend to help the nurse when her working days were done.

It is an interesting fact in the history of nursing that even Miss Nightingale herself was inclined to throw cold water on his scheme. In a remarkable letter preserved among Sir Henry's papers, Miss Nightingale pointed out how difficult the success of such a Fund would be, and implied, though she did not state it with complete frankness, that no Pension Fund could succeed because the nurses were never likely to be able to save anything out of their salaries; and therefore could not insure their future. It did not seem to have occurred to Miss Nightingale that a guarantee fund was the first essential, and that Sir Henry would not project the Pension Fund until this preliminary endowment had been raised. When the scheme, however, was announced the names of four donors of £5,000 each were announced with it, and the praise with which the Press welcomed the scheme was chiefly excited by the energy and foresight which had created a guarantee fund to ensure its success. These facts place Sir Everard Hambro's resolution in its proper setting, and Sir Henry's family cannot but be grateful to the nurses and to Miss Skillman and Miss Fisher who generously voiced their thanks.

We note that Sir Everard has received, he says, "several suggestions how nurses could best perpetuate Sir Henry's memory." He did not say what these suggestions were, but himself proposed that they could not better do so than by "pushing forward the Fund." This suggestion is somewhat vague. It remains to be seen if the nurses are really anxious for a memorial. The decision rests with them. But if any memorial is to be raised at all, let it be the expression of a strong desire, with a definite idea behind it. Half measures were not characteristic of the man, who in the absence of any definite wish on the part of the rank and file, would prefer to live in the endurance of his work for nurses. This work was to lay the financial foundation of the great fund which year by year befriends the hospital nurse in old age, sickness, and adversity.

